# CNS aspergillosis in a patient with Crohn’s disease on immunosuppressants: a case report

**DOI:** 10.4076/1757-1626-2-6376

**Published:** 2009-06-16

**Authors:** Shreyansh Shah, Peyman Shirani, Heike Schmolck, William C Young, Paul E Schulz

**Affiliations:** 1 Department of Neurobiology and Anatomy, University of Texas Health Science Center at Houston6431 Fannin Street, Houston, TX 77030USA; 2 Department of Neurology, NB-302, Baylor College of MedicineOne Baylor Plaza, Houston, TX 77030USA; 3 Mercy Ruan Neurology ClinicDes Moines, IA 50314USA

## Abstract

Fungal infections of the central nervous system are an uncommon cause of rapid decline in consciousness. We describe the case of central nervous system aspergillosis in a patient on immunosupressants whose clinical course highlights the need for an aggressive approach to diagnosis.

## Case presentation

**Figure 1:**
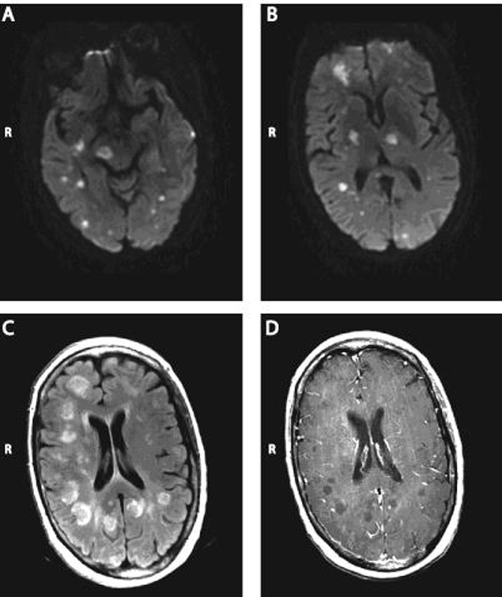
Axial MR images demonstrating diffusion restriction **(A & B)**, hyperintensities on FLAIR images **(C)**, and hypointensities on T1 images **(D)**.

A 53-year-old right-handed Caucasian woman with a history of Crohn’s disease for four decades presented with pneumonia and a change in mental status. She was taking prednisone daily for a recent Crohn’s exacerbation. Methotrexate was added 2 weeks prior. On physical examination, she had hypotension, tachycardia and thrombocytopenia. She was comatose and responses to painful stimuli were decreased on left side. A CT scan of the brain showed multiple areas of hypodensity. A brain MRI (Figure 1) showed numerous foci of T1 hypointensity and T2 hyperintensity in the periventricular, subcortical and deep white matter, including the gray-white junction. There were also lesions in the basal ganglia, thalami, pons and cerebellum that showed diffusion restriction. The distribution and properties of these radiological images were suggestive of septic emboli. She had an extensive left upper lobe consolidation on chest X-Ray. Bronchoscopy revealed aspergillus, which lead to a diagnosis of CNS aspergillosis. Despite antifungal therapy she succumbed to her disease and the decision was made to withdraw care in light of her poor neurological status.

## Discussion

Aspergillus is a saprophytic, opportunistic fungus that can infect humans, especially immunocompromised hosts [1]. The primary portal of entry for aspergillus is the respiratory tract. From there, it secondarily infects the brain via hematogenous spread. In some cases, it can also result from penetrating trauma or extension of infection from the mastoid air sinuses [2].

CNS aspergillosis should be considered in patients presenting with the acute onset of focal neurologic deficits, especially in immunocompromised hosts. The most frequent symptoms are headache, vomiting, convulsion, hemiparesis, fever, cranial nerve deficits, paralysis and sensory impairment of varying degrees. Since aspergillus can form mycotic aneurysms, it can lead to subarachnoid hemorrhage and meningeal signs. The propensity of the fungus to invade blood vessels may lead to extensive necrosis or intracranial bleeding [3].

The MRI in CNS aspergillosis typically shows infarction or abscesses in multiple brain areas, including the basal ganglia and thalami [4]. Although the mortality rate in CNS aspergillosis approaches 95% [5], recent reports suggest that early initiation of antifungal therapy with neurosurgical intervention can improve outcomes [6].

## List of abbreviations

CT: Computerized tomography
CNS: Central nervous system
MRI: Magnetic resonance imaging
